# Leveraging the enrichment analysis from a genome-wide association study against epilepsy—focusing on the role of tryptophan catabolites pathway in patients with drug-resistant epilepsy

**DOI:** 10.3389/fnut.2025.1539145

**Published:** 2025-08-06

**Authors:** Alice Y. W. Chang, Chin-Wei Huang, Ping-Lin Tsai, Chun-An Liang, Wei Chen Liao, Tzu-Fun Fu, Hui Hua Chang

**Affiliations:** ^1^Department of Physiology, College of Medicine, National Cheng Kung University, Tainan, Taiwan; ^2^Institute of Basic Medical Sciences, College of Medicine, National Cheng Kung University, Tainan, Taiwan; ^3^Department of Neurology, National Cheng Kung University Hospital, College of Medicine, National Cheng Kung University, Tainan, Taiwan; ^4^Institute of Clinical Pharmacy and Pharmaceutical Sciences, College of Medicine, National Cheng Kung University, Tainan, Taiwan; ^5^Department of Pharmacy, Kaohsiung Veterans General Hospital, Kaohsiung, Taiwan; ^6^Department of Medical Laboratory Science and Biotechnology, College of Medicine, National Cheng Kung University, Tainan, Taiwan; ^7^School of Pharmacy, College of Medicine, National Cheng Kung University, Tainan, Taiwan; ^8^Department of Pharmacy, National Cheng Kung University Hospital, College of Medicine, National Cheng Kung University, Tainan, Taiwan; ^9^Department of Pharmacy, National Cheng Kung University Hospital, Dou-Liou, Yunlin, Taiwan

**Keywords:** epilepsy, drug-resistant epilepsy, genome-wide association study, pharmacogenomics, tryptophan catabolites, multivitamin supplementation

## Abstract

**Background:**

Drug-resistant epilepsy (DRE) is a chronic neurological disorder with somatic impacts and an increased risk of psychiatric comorbidities and cognitive impairment. Previous studies suggested that genomic variants could contribute to the high interindividual variability in epilepsy and in its treatment response, but it remains unclear. Here, we aimed to perform genome-wide association study (GWAS), leverage the enrichment analysis of the genomic variants, and provide the potential molecular signature profiles. Moreover, we investigated the potential role of molecular signature profiles, as exemplified by tryptophan catabolites (TRYCATs), in DRE patients.

**Methods:**

We used data from the Taiwan Biobank to perform a GWAS and identified enrichment pathways through the functional database Reactome. To validate the results, we enrolled community-dwelling controls and DRE patients. The levels of TRYCATs were determined using liquid chromatography–tandem mass spectrometry. In addition, we compared the levels of TRYCATs between the controls and DRE patients at baseline and after 6-month multivitamin supplementation. Seizure frequency was defined as the number of episodes per 28 days in DRE patients.

**Results:**

Using GWAS and enrichment analysis of genomic data, we obtained candidate genes implicated in mechanisms and molecular signature profiles against epilepsy, such as the TRYCATs pathway. To validate the molecular signature from enrichment analysis, we further examined whether the TRYCATs pathway was associated with the pathophysiology of epilepsy and treatment outcome in DRE patients. We found that DRE patients had significantly lower levels of TRYCATs (tryptophan, serotonin, 3-indole acetic acid, 3-indoleperopionic acid, kynurenine, and kynurenic acid) than the controls. Additionally, changes in the balance of the TRYCATs pathway were noted in DRE patients treated with 6-month multivitamin supplementation. Furthermore, the change levels of TRYCATs were correlated with seizure frequency in the DRE patients during multivitamin supplementation.

**Conclusion:**

The TRYCATs pathway plays an important role in the pathophysiology of epilepsy and is involved in the multivitamin-mediated physiological alterations in DRE patients. Therefore, the balance of TRYCATs might be a new biomarker and therapeutic strategy for epilepsy.

## 1 Introduction

Epilepsy is a chronic neurological disorder resulting from malfunctioning nerve cell activity in the brain, which is characterized by recurrent episodic attacks and epileptic seizures and causes somatic and psychiatric impacts (1). Globally, 6.4 per 1,000 individuals have active epilepsy, and the lifetime prevalence is 7.6 per 1,000 (2, 3). Epilepsy affects different aspects of daily life, ranging from sleep quality, studying or working efficiency to daily safety. A high hospitalization rate, comorbidity and mortality are reported in all age groups with epilepsy (4). Although there are expanding lists of available antiepileptic drugs (AEDs), approximately 30% of people who continue to have seizures after adequate trials of two AED treatments develop drug-resistant epilepsy (DRE) (5, 6). In addition, patients with DRE have increased rates of medical and psychiatric comorbidities that could complicate epilepsy management, contribute to decreased health-related quality of life (HRQoL) (7), increase health-care costs, and even shorten the lifespan (4, 8).

Genomic variants could contribute to the high interindividual variability in treatment response or adverse effects (such as weight gain and altered lipid profiles) to AEDs (9–13). For epilepsy, the clinical observation that therapeutic response to the first AED predicts response to subsequent AEDs (14) supports the presence of individual effects on broad treatment response. Twin studies suggest that such individual effects on the outcome of treated epilepsy are mediated by epilepsy genomic susceptibility factors (9). With construction for the entire human genome based on high-throughput single nucleotide polymorphisms (SNPs), the detection of specific DNA sequences affecting responses to drugs can now be made possible (15, 16). Genome-wide association studies (GWAS) could provide a conceptual framework in the search for variants underlying mechanisms against epilepsy and AED responses (17–20). Furthermore, pathway enrichment analysis from post-GWAS analysis could leverage the joint effect of common variants in pathways that can be putatively modulated by known pharmacological compounds or molecular signaling pathways (21–23). Therefore, we expect to identify possible seizure-related loci and mechanisms using a pharmacogenomics approach.

However, GWASs examining Taiwanese epilepsy are limited. In this study, therefore, we aimed to investigate the association between genetic polymorphisms and epilepsy in the Taiwanese population, and we obtained candidate genes implicated in mechanisms against epilepsy through the GWAS approach. Moreover, we investigated the molecular signature from enrichment analysis of genomic data and validated the results in DRE patients who received add-on multivitamin therapy (24). The results of the current study revealed that the tryptophan catabolites (TRYCATs) pathway was associated with the pathophysiology of epilepsy and correlated with seizure frequency after multivitamin supplementation in DRE patients.

## 2 Materials and methods

### 2.1 Subjects from the Taiwan Biobank to perform GWAS and post GWAS analyses

The Taiwan Biobank is an available large-scale Taiwanese population-based cohort. The use of data complies with the specifications of Academia Sinica Medical Research Ethical Institutional Review Board and Academia Sinica Taiwan Biobank Database Ethical Governing Committee. We extracted subjects with recorded epilepsy from all subjects and 1:3 matched a control (nonepilepsy) group by age ±5 y/o and gender. The subject surveys, physical examination data, and whole-genome genotype information from the Taiwan Biobank database were obtained. DNA was extracted from venous blood samples, and the whole genome genotype profiles of each subject were genotyped using the Axiom TWB v1.0 array (653,291 SNPs) and/or the Axiom TWB v2.0 array (710,525 SNPs) (Thermo Fisher Scientific) by the National Center for Genome Medicine utilizing the GeneTitan Multi-Chanel instrument automated operating platform.

### 2.2 Analyses of GWAS

#### 2.2.1 SNPs quality control and imputation

The sample and SNP quality control were performed on SNP & Variation Suite™ (Version 8.8.3) (Golden Helix, Inc., Bozeman, MT, USA). Principal component analyses (PCA) were performed to correct ancestry diversity. Sex check analysis, defined heterozygosity < 0.02 as males, was performed. The parameters for linkage disequilibrium (LD) pruning of the samples were referred to the default in PLINK, as 50 for window size, 5 for window increment, r2 for LD statistic, 0.5 for LD threshold, and CHM for LD Computation Method (25). The cryptic relatedness was examined by pairwise identity-by-descent (IBD) estimation (PI = P (Z = 1)/2 + P (Z = 2), PI < 0.1875). All of the subjects meeting the following criteria were excluded: (i) SNPs with call rate < 0.95, (ii) Hardy–Weinberg equilibrium (HWE) < 1e-6, (iii) and minor allele frequency (MAF) < 1%.

To match the two different arrays, we first transformed the genome version in array 2 (hg38) to the same as array 1 (hg19) by an assembly converter (liftover) in Ensembl (26). The samples and SNPs that passed the previous quality control criteria were then imputed using the 1,000 Genome project Phase 3 panel (Filter 1% AF) (total 13,997,073 markers were read from the reference panel) and conducted on Beagle 4.1 (27, 28) in SNP & Variation Suite ™. We set the genotype to missing if the genotype probability was less than 0.85, and the general parameters were window size: 50,000 and overlap: 3000. For the phasing and imputation algorithm, the parameters were set as follows: Phasing Iterations = 5, Max Cluster Size in CM = 0.005, Effective Population Size = 1,000,000, Allele Miscall Rate = 0.0001. After imputing the two arrays separately, we performed quality control on the markers. The filtration criteria of imputation quality were genotype probability < 0.9 and dosage *R*-squared < 0.8. Then, we merged the two arrays and performed another cryptic relatedness test. Finally, SNPs with call rate < 0.9, MAF < 0.01 and HWE < 1*e^−6^ based on controls were excluded from further studies.

#### 2.2.2 Association tests and models

For autosomal chromosomes, logistic regression methods were used to test for association between epilepsy and nonepilepsy for each variant, and the covariates were selected by the PCA on clinical indices and comorbidities of those subjects. The estimated regression coefficient for each SNP (denoted by *β*) and standard error (SE) were calculated for the minor alleles. LD between pairs of SNPs was carried out employing expectation maximization (EM). The Manhattan and quantile-quantile plots corresponding to the results were calculated and implemented on the SNP and Variation Suite ™.

### 2.3 Bioinformatics of post GWAS analyses

#### 2.3.1 SNP annotation and gene-based association analysis

The SNP annotation was based on the Ensembl (26, 29) and RefSeq (30) databases. In addition, we performed gene-set association analysis using our GWAS results. Gene-based association analysis was performed using the Gene-based Association Test using Extended Simes procedure (GATES) (31) method, which is modeled in KGG software, a systematic biological knowledge-based mining system. The gene mapping information was based on GENCODE databases (32). The defined length of the extended gene region was ±10 kb for each gene. LD was adjusted based on EAS genotype data from the 1,000 Genome Project Phase 3 reference population (33) in the analyses. A Benjamini and Hochberg (34) false discovery rate (FDR) correction was applied to control for the multiple tests. The gene-based suggestive threshold was set at corrected *p* < 0.05.

#### 2.3.2 Gene differential tissue expression analysis and functional pathway enrichment analysis

We further analyzed the gene expression of the top gene sets. The gene expression heatmap and tissue differential expression were generated using the GTEx V8 (35) database. To gain insights into the functions of the identified genes, we tested the probability of the identified genes being involved in particular biological pathways. We identified significantly enriched pathways using the functional pathway database Reactome (36).

### 2.4 Subjects recruited from outpatients

The research protocol was approved by the Ethical Committee for Human Research at the National Cheng Kung University Hospital (IRB No. A-ER-105-489), and written informed consent was obtained from each subject before any procedures were performed. This study was conducted in accordance with the Declaration of Helsinki. The participants (aged 20–65 years) were enrolled consecutively by a trained neurologist and diagnosed with DRE as described previously (24). Briefly, the inclusion criteria for refractory epilepsy were (1) a diagnosis of epilepsy and (2) the failure of two or more antiepileptic drugs (AEDs) and the occurrence of one or more seizures per month over 18 months (37, 38). Participants (1) undergoing surgery for epilepsy, (2) with an organic mental disorder, mental retardation, dementia, or other diagnosed neurological illness, (3) with a surgical condition or a major physical illness, and (4) who were pregnant or breastfeeding were excluded from our study. In addition, the participants received add-on multivitamin therapy for 6 months and certain DRE patients showed a significant reduction in seizure frequency (24). Briefly, participants received a daily dose of vitamin B6 (100 mg), vitamin B9 (5 mg), vitamin D (1,000 IU), vitamin E (400 IU), and coenzyme Q10 (100 mg) (24). Seizure frequency was defined as the number of episodes per 28 days based on medical records from neurologists and was used to assess the severity of the disease. To compare the level of TRYCATs with patients with epilepsy, we also recruited controls from the community through an advertisement. They recruited subjects without neurological illness, psychiatric illness, severe physical illness (such as cardiovascular diseases and cancers), and a past history of inflammatory diseases (such as type 2 diabetes mellitus and hyperlipidemia). In addition, the body weight and height of each subject were measured, and the BMI (kg/m^2^) was calculated accordingly.

### 2.5 Measurements

Blood samples were collected from the antecubital vein in heparinized plain tubes after fasting for 8 to 12 h. Serum and plasma were separately isolated from whole blood by centrifugation at 1500 rpm for 15 min at 4°C and were then immediately stored at −80°C.

### 2.6 Tryptophan catabolites

The TRYCATs levels (tryptophan (TRP), 5-hydroxytryptophan (5-HTP), serotonin, kynurenine (KYN), kynurenic acid (KYNA), 3-indoleperopionic acid (IPA), and 3-indole acetic acid (IAA)) were measured by liquid chromatography–tandem mass spectrometry (LC–MS/MS) (Agilent 6,470 triple quadrupole LC/MS system) at baseline and after 1, 3, and 6 months of add-on multivitamin therapy. The levels of TRYCATs were calculated and corrected from the standard calibration curve.

### 2.7 Statistical analysis

Statistical analysis was performed using Statistical Package for Social Sciences version 18.0 for Windows/Mac (SPSS Inc., Chicago, IL, USA). All demographic and clinical characteristics of the subjects were expressed either as numbers and percentages for categorical variables or as the mean ± standard deviation for continuous variables. Student’s *t-*test, one-way analysis of variance (ANOVA), and chi-square (χ^2^) tests were used to assess the differences in characteristics between groups. Correlations were assessed by Spearman’s correlation test. The generalized estimating equations (GEE) were employed to analyze the repeated measurements of TRYCATs over time (baseline, 1, 3, and 6 months) and to assess the interaction between treatment (multivitamin supplementation) and time. Covariates included in the GEE models were age and baseline metabolite levels, which were chosen based on the significant differences between groups. The level of significance was set at 0.05 for two-sided tests.

## 3 Results

### 3.1 GWAS and post GWAS analysis from databases of the Taiwan Biobank

**Figure 1:**
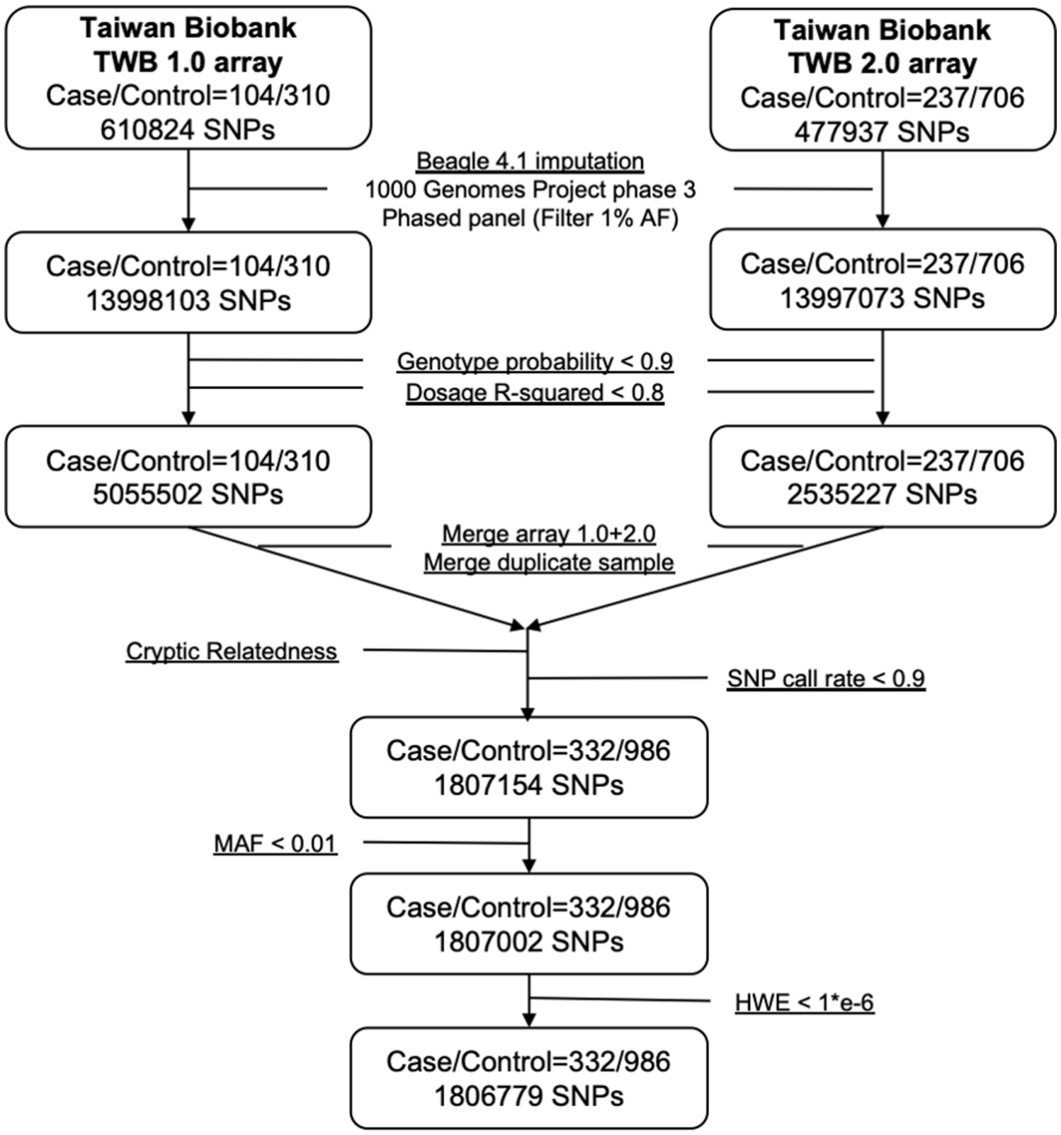
Flowchart of imputation and quality control when combining Axiom TWB 1.0 and TWB 2.0 array.

**Figure 2:**
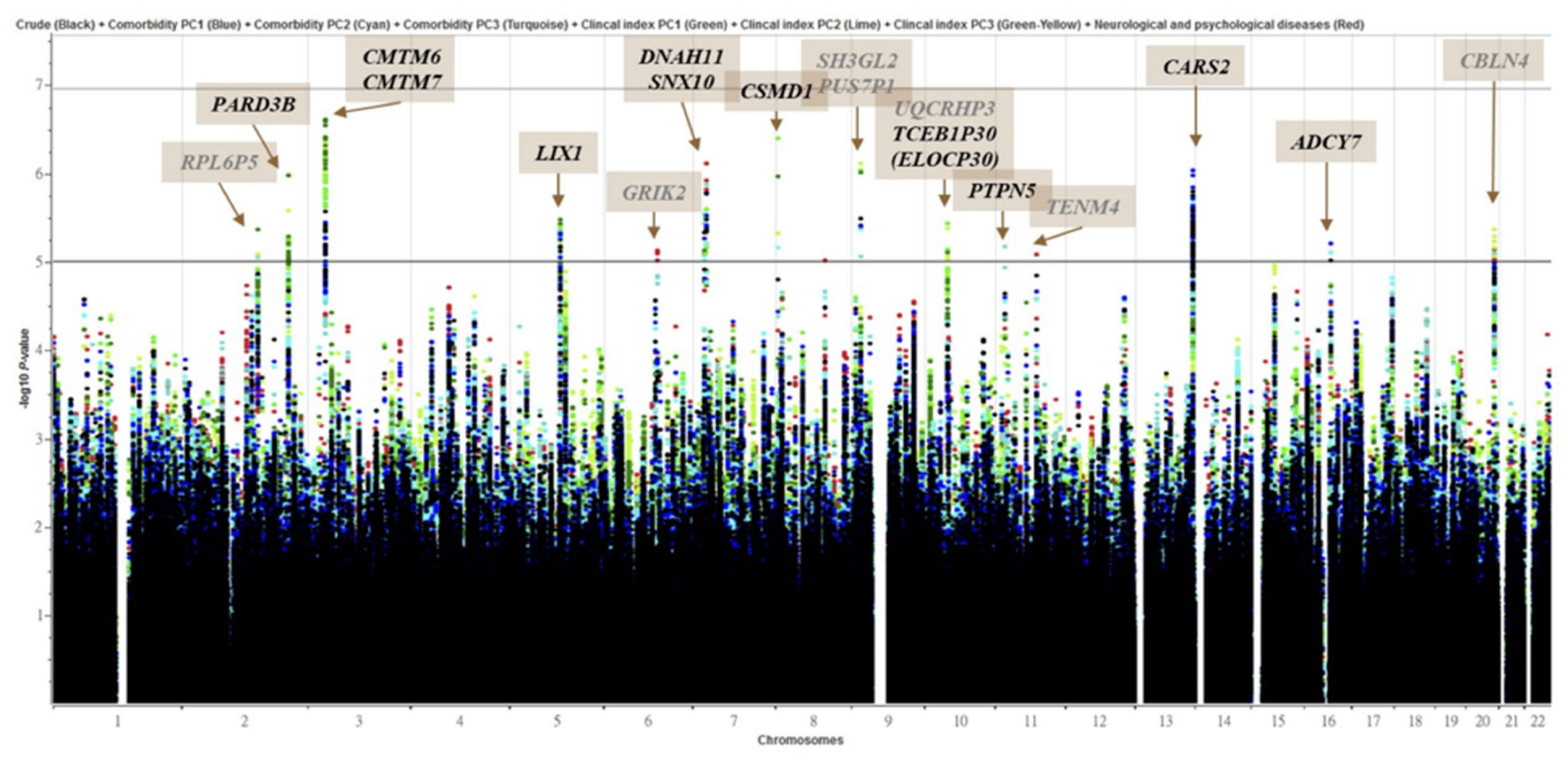
Manhattan plot of genome-wide association analyses for epilepsy (*n* = 332) and control (*n* = 986). *X*-axis shows chromosomal positions. *Y*-axis shows –log10 *p*-values from logistic regression. The horizontal black, iron gray and gray lines indicate the suggestive (1 × 10^−5^), and genome-wide significance (*p* = 0.2 (1.11 × 10-7)), (*p* = 0.05 (2.77 × 10-8)) *p*-value thresholds, respectively. Genes overlapped (Black) or nearby (Gray) significant locus were highlighted. The plot combined 8 models which were marked with different colored dots.

The 341 epilepsy and 1,023 nonepilepsy subjects (randomly extracted from 95,252) were 1:3 age-gender-matched from databases of the Taiwan Biobank, appraising a prevalence of 357.99 per 100,000 persons. Figure 1 shows the flow diagram of the whole genome analysis for epilepsy in the Taiwan Biobank database, and 332 epilepsy and 986 nonepilepsy subjects have genomic profiles after SNPs quality control. The demographic characteristics of the subjects from the Taiwan Biobank databases are shown in Table 1, and the comorbidities of the subjects are shown in Table 2. Because there were significant differences in the levels of clinical indices and the percentage of comorbidities, we performed principal component analysis (PCA) to obtain signature factors for further adjusting in GWAS analyses. After conducting PCA, the top three component factors of clinical indices and comorbidities were selected (Supplementary Tables S1, S2). Furthermore, the GWAS results showed significant loci in the crude model (Figure 2) and in the models with adjustment for different components of PCA (Supplementary Table S3).

**Table 1:** Clinical indices of physical examinations and metabolic indices in the Taiwan Biobank.

Characteristics	Epilepsy (*n* = 332)	Non-epilepsy (*n* = 986)	Comparison		
	Mean ± SD	Mean ± SD	*t*/χ^2^	95% CI	*p*-value
Physical examination					
Body height, cm	163.55 ± 8.37	163.00 ± 8.49	1.02	(−0.50–1.60)	0.307
Body weight, kg	67.80 ± 14.99	64.75 ± 12.59	3.34	(1.26–4.85)	<0.001^***^
BMI, kg/m^2^	25.22 ± 4.51	24.24 ± 3.55	3.59	(0.44–1.51)	<0.001^***^
Body fat rate, %	28.19 ± 7.83	27.08 ± 7.00	2.23	(0.13–2.08)	0.027^*^
Waist circumference, cm	86.12 ± 12.16	83.82 ± 9.52	3.14	(0.86–3.74)	0.002^**^
Hip circumference, cm	97.86 ± 8.31	96.47 ± 6.54	2.78	(0.41–2.38)	0.006^**^
Waist/hip ratio	0.88 ± 0.07	0.87 ± 0.06	2.19	(0.00–0.02)	0.029^*^
BAI	28.94 ± 4.59	28.51 ± 3.92	1.54	(−0.12–0.98)	0.124
Systolic pressure, mmHg	118.86 ± 17.54	113.97 ± 17.86	4.35	(2.69–7.10)	<0.001^***^
Diastolic pressure, mmHg	72.94 ± 11.11	70.58 ± 11.41	3.29	(0.95–3.76)	0.001^**^
Heart beats, per 30 s	35.22 ± 4.83	34.98 ± 4.35	0.85	(−0.31–0.80)	0.394
Bone index					
Stiffness index	90.74 ± 19.76	94.58 ± 17.25	−3.15	(−6.23--1.45)	0.002^**^
Young-adult, %	92.93 ± 20.52	96.92 ± 17.91	−3.15	(−6.47--1.50)	0.002^**^
*T*-score	−0.62 ± 1.84	−0.27 ± 1.61	−3.11	(−0.57--0.13)	0.002^**^
Age-matched, %	108.58 ± 21.49	113.32 ± 19.15	−3.57	(−7.36--2.13)	<0.001^***^
*Z* score	0.67 ± 1.66	1.01 ± 1.48	−3.36	(−0.55--0.14)	<0.001^***^
Blood count					
WBC, 10^3/uL	5.81 ± 1.70	5.95 ± 1.58	−1.41	(−0.34–0.06)	0.159
RBC, 10^6/uL	4.76 ± 0.57	4.79 ± 0.51	−0.94	(−0.10–0.04)	0.349
Hb, g/dL	13.82 ± 1.77	14.07 ± 1.53	−2.24	(−0.46--0.03)	0.025^*^
Hct, %	41.39 ± 4.60	44.02 ± 4.46	−9.23	(−3.18--2.07)	<0.001^***^
PLT, 10^3/uL	233.50 ± 62.16	236.76 ± 57.52	−0.88	(−10.55–4.03)	0.38
Sugar profile					
HbA1c, %	5.80 ± 0.87	5.67 ± 0.76	2.51	(0.03–0.22)	0.012^*^
AC glucose, mg/dl	96.41 ± 22.90	95.14 ± 16.97	0.93	(−1.41–3.95)	0.353
Lipid profile					
Cholesterol, mg/dl	192.60 ± 34.74	192.19 ± 34.99	0.19	(−3.92–4.75)	0.852
HDL, mg/dl	53.80 ± 15.34	54.55 ± 13.92	−0.83	(−2.53–1.02)	0.406
LDL, mg/dl	117.20 ± 30.98	121.26 ± 32.29	−2.01	(−8.03--0.09)	0.045^*^
TG, mg/dl	123.93 ± 87.96	122.34 ± 86.97	0.29	(−9.23–12.42)	0.772
Cholesterol / HDL ratio	3.80 ± 1.07	3.71 ± 1.05	1.4	(−0.04–0.22)	0.163
LDL / HDL ratio	2.35 ± 0.86	2.36 ± 0.82	−0.15	(−0.11–0.10)	0.882
TG / HDL ratio	2.75 ± 2.66	2.58 ± 2.52	1.04	(−0.15–0.49)	0.298
Liver function profile					
Bilirubin, mg/dL	0.59 ± 0.26	0.71 ± 0.28	−6.65	(−0.15--0.08)	<0.001^***^
Albumin, g/dL	4.51 ± 0.25	4.63 ± 0.27	−7.1	(−0.15--0.09)	<0.001^***^
AST, U/L	25.56 ± 12.92	23.11 ± 12.76	3.03	(0.86–4.04)	0.003^**^
ALT, U/L	24.04 ± 17.47	25.60 ± 21.14	−1.22	(−4.09–0.95)	0.222
AST/ALT ratio	1.29 ± 0.55	1.07 ± 0.43	6.59	(0.15–0.28)	<0.001^***^
AFP, ng/mL	2.94 ± 4.54	2.95 ± 1.87	−0.06	(−0.52–0.49)	0.952
γ-GT, U/L	40.21 ± 50.90	25.81 ± 28.94	4.9	(8.63–20.17)	<0.001^***^
Kidney function profile					
BUN, mg/dL	12.69 ± 4.30	13.20 ± 4.01	−1.99	(−1.02--0.01)	0.047^*^
Creatinine, mg/dL	0.75 ± 0.24	0.81 ± 0.44	−2.15	(−0.10–0.00)	0.032^*^
Uric acid, mg/dL	5.54 ± 1.70	5.65 ± 1.44	−1	(−0.31–0.10)	0.319
microALB, mg/L	39.88 ± 208.40	27.36 ± 136.41	1.02	(−11.63–36.68)	0.309

Data are presented as the mean ± SD or number (percentage). BMI, body mass index; BAI, body adiposity index; WBC, white blood cell; RBC, red blood cell; Hb, hemoglobin; Hct, hematocrit; PLT, blood platelet; HbA1c, glycated hemoglobin A1c; ACglucose, glucose ante cibum; HDL, high-density lipoprotein cholesterol; LDL, low-density lipoprotein cholesterol; TG, triglyceride; AST, aspartate aminotransferase; ALT, alanine aminotransferase; AFP, alpha-fetoprotein; γ-GT, gamma-glutamyl transpeptidase; BUN, blood urea nitrogen; microALB, microalbumin. ^*^p < 0.05; ^**^p < 0.01; ^***^p < 0.001.

**Table 2:** The comorbidities of epilepsy and nonepilepsy in the Taiwan Biobank.

Comorbidities	Epilepsy (*n* = 332)	Non-epilepsy (*n* = 986)	Comparison	
	*n* (%)	*n* (%)	χ^2^	*p*-value
Neurology and psychiatry				
Depression	34 (10.2)	28 (2.8)	30.88	<0.001^***^
Bipolar disorder	12 (3.6)	4 (0.4)	21.59	<0.001^***^
Postpartum depression	5 (1.5)	3 (0.3)	6.04	0.014^*^
Obsessive compulsive disorder	3 (0.9)	2 (0.2)	3.28	0.070
Alcoholism or drug addition	3 (0.9)	0 (0.0)	9.02	0.003^**^
Schizophrenia	10 (3.0)	2 (0.2)	21.98	<0.001^***^
Paroxysmal hemicrania	24 (7.2)	23 (2.3)	17.65	<0.001^***^
Multiple sclerosis	0 (0.0)	0 (0.0)	–	–
Parkinson’s disease	3 (0.9)	0 (0.0)	9.02	0.003^**^
Dementia	3 (0.9)	0 (0.0)	9.02	0.003^**^
Vertigo	30 (9.0)	34 (3.4)	17.16	<0.001^***^
Cardiovascular diseases				
Valve heart disease	14 (4.2)	24 (2.4)	2.93	0.087
Coronary artery disease	8 (2.4)	18 (1.8)	0.47	0.493
Arrhythmia	22 (6.6)	34 (3.4)	6.36	0.012^*^
Cardiomyopathy	7 (2.1)	0 (0.0)	21.11	<0.001^***^
Congenital heart disease	1 (0.3)	2 (0.2)	0.11	0.739
Other heart disease	0 (0.0)	2 (0.2)	0.67	0.414
Apoplexy	12 (3.6)	1 (0.1)	31.72	<0.001^***^
Hyperlipidemia	42 (12.6)	64 (6.4)	13.13	<0.001^***^
Hypertension	55 (16.5)	96 (9.6)	11.85	<0.001^***^
Diabetes	24 (7.2)	48 (4.8)	2.82	0.093
Aches				
Osteoporosis	18 (5.4)	65 (6.5)	0.52	0.472
Arthritis	23 (6.9)	46 (4.6)	2.70	0.101
Gout	16 (4.8)	43 (4.3)	0.148	0.701
Gastroenterology, hepatology and nephrology				
Peptic ulcer	58 (17.4)	167 (16.7)	0.09	0.768
Gastroesophageal reflux	45 (13.5)	67 (6.7)	15.03	<0. 001^***^
Irritable bowel syndrome	13 (3.9)	20 (2.0)	3.74	0.053
Liver gall stone	16 (4.8)	40 (4.0)	0.40	0.528
Kidney stone	24 (7.2)	65 (6.5)	0.20	0.657
Renal failure	1 (0.3)	2 (0.2)	0.11	0.739
Ophthalmology				
Cataract	24 (7.2)	74 (7.4)	0.02	0.904
Glaucoma	6 (1.8)	19 (1.9)	0.01	0.907
Xerophthalmia	34 (10.2)	91 (9.1)	0.57	0.551
Retinal detachment	6 (1.8)	14 (1.4)	0.27	0.603
Floaters	39 (11.7)	116 (11.6)	0.00	0.961
Blind	4 (1.2)	0 (0.0)	12.04	<0.001^***^
Color blind	3 (0.9)	6 (0.6)	0.34	0.562
Other eye disease	19 (5.7)	33 (3.3)	3.84	0.050^*^
Others				
Allergic	40 (12.0)	63 (6.3)	11.40	<0.001^***^
Asthma	20 (6.0)	31 (3.1)	5.72	0.017^*^
Emphysema or bronchitis	6 (1.8)	9 (0.9)	1.82	0.177

^*^p < 0.05; ^**^p < 0.01; ^***^p < 0.001.

**Figure 3:**
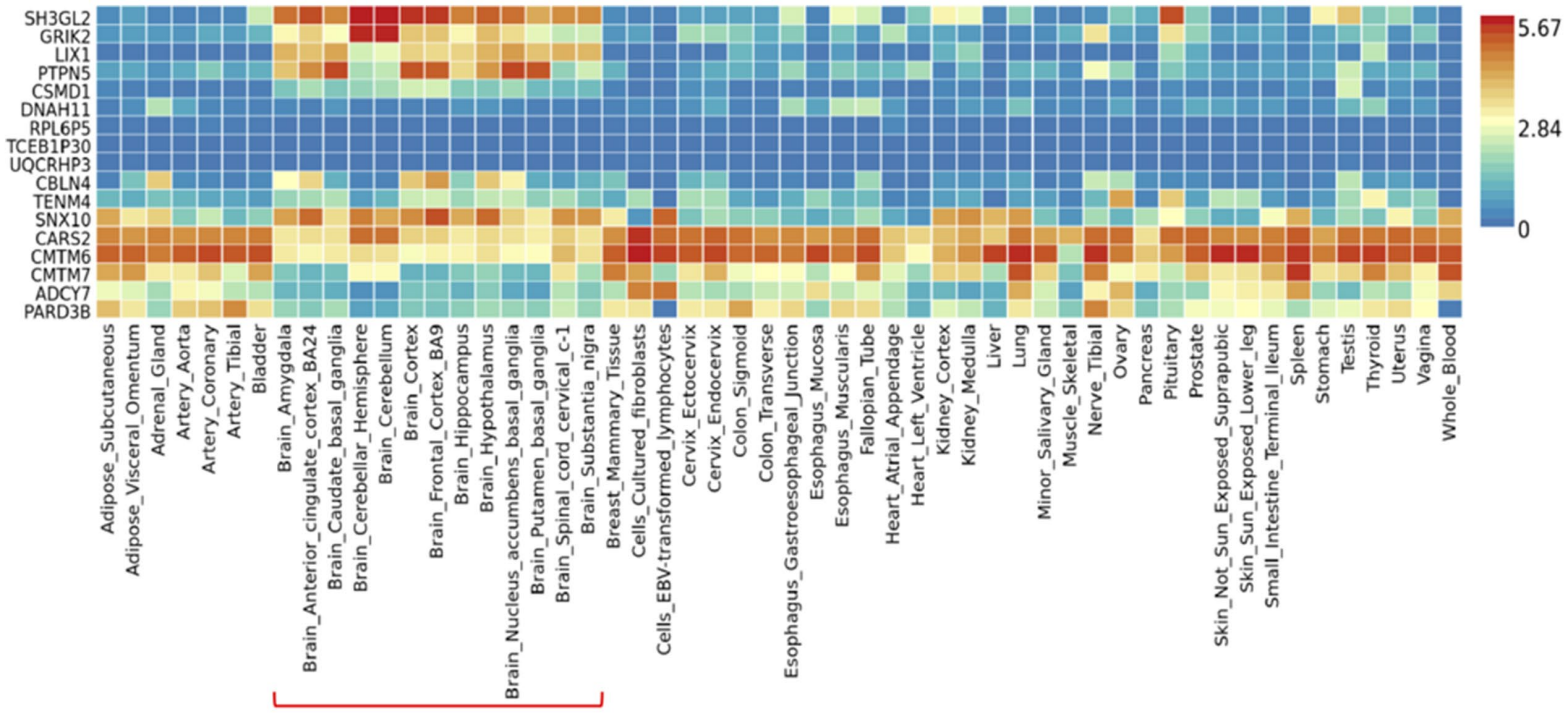
Gene expression heatmap of 54 tissues in GTEx v8. Data was presented as average of log2 transformed expression value (TPM) per tissue type. Brain regions were highlighted in the red line.

To investigate the differential tissue expression of the top genes, the gene expression analysis was shown in a heatmap using the GTEx database (Figure 3). The results showed that *SH3GL2*, *GRIK2*, *LIX1*, *PTPN5*, *SNX10*, *CARS2*, and *CMTM6* were expressed at higher levels in brain regions than other genes. In addition to leveraging the genomic profiles from GWAS data, we performed functional pathway enrichment analysis using the Reactome database. The results showed that most pathways were related to neurotransmitter receptors and postsynaptic signal transmission, protein kinase A (PKA) activation, and interleukin signaling (Table 3). Furthermore, to validate the molecular signature from enrichment analysis, we examined whether the TRYCATs pathway was associated with the pathophysiology of epilepsy and treatment outcome in DRE patients who received add-on multivitamin therapy.

**Table 3:** Top 25 Reactome pathways from the enrichment analysis of genomic data.

Pathway ID	Category	Pathway name	Entities				Reactions		Overlapped gene
			Found/Total	Ratio	*p* value	FDR	Found/Total	Ratio	
R-HSA-451308	Calcium and sodium	Activation of Ca-permeable kainate receptor	2/13	8.96E-04	1.51E-04	0.007339	2/2	1.57E-04	*GRIK2*
R-HSA-451306	Calcium and sodium	Ionotropic activity of kainate receptors	2/14	9.65E-04	1.75E-04	0.007339	4/4	3.15E-04	*GRIK2*
R-HSA-451326	Calcium and sodium	Activation of kainate receptors upon glutamate binding	2/34	0.002343	0.001014	0.023828	4/6	4.72E-04	*GRIK2*
R-HSA-9008059	Interleukin	Interleukin-37 signaling	2/36	0.00248	0.001135	0.023828	1/14	0.001102	*PTPN5*
R-HSA-112314	Neurotransmitter	Neurotransmitter receptors and postsynaptic signal transmission	3/232	0.015985	0.003798	0.060767	9/109	0.008583	*GRIK2; ADCY7*
R-HSA-451307	Calcium and sodium	Activation of Na-permeable kainate receptors	1/5	3.44E-04	0.006867	0.094675	2/2	1.57E-04	*GRIK2*
R-HSA-112315	Neurotransmitter	Transmission across chemical synapses	3/352	0.024252	0.01194	0.094675	9/161	0.012677	*GRIK2; ADCY7*
R-HSA-446652	Interleukin	Interleukin-1 family signaling	2/163	0.011231	0.020955	0.094675	1/79	0.00622	*PTPN5*
R-HSA-170660	PKA activation and energy transform	Adenylate cyclase activating pathway	1/17	0.001171	0.023167	0.094675	4/4	3.15E-04	*ADCY7*
R-HSA-170670	PKA activation and energy transform	Adenylate cyclase inhibitory pathway	1/18	0.00124	0.024514	0.094675	5/5	3.94E-04	*ADCY7*
R-HSA-177504	Neurotransmitter	Retrograde neurotrophin signaling	1/18	0.00124	0.024514	0.094675	2/3	2.36E-04	*SH3GL2*
R-HSA-8875360		InlB-mediated entry of *Listeria monocytogenes* into host cell	1/19	0.001309	0.025859	0.094675	2/8	6.30E-04	*SH3GL2*
R-HSA-112316	Neurotransmitter	Neuronal system	3/498	0.034312	0.029756	0.094675	9/214	0.01685	*GRIK2; ADCY7*
R-HSA-164378	PKA activation and energy transform	PKA activation in glucagon signaling	1/23	0.001585	0.031221	0.094675	1/2	1.57E-04	*ADCY7*
R-HSA-163615	PKA activation and energy transform	PKA activation	1/23	0.001585	0.031221	0.094675	1/4	3.15E-04	*ADCY7*
R-HSA-6807004	PKA activation and energy transform	Negative regulation of MET activity	1/25	0.001722	0.033892	0.094675	2/10	7.87E-04	*SH3GL2*
R-HSA-111931	PKA activation and energy transform	PKA-mediated phosphorylation of CREB	1/26	0.001791	0.035224	0.094675	1/7	5.51E-04	*ADCY7*
R-HSA-8876384	Others	*Listeria monocytogenes* entry into host cells	1/27	0.00186	0.036555	0.094675	2/13	0.001024	*SH3GL2*
R-HSA-182971	Cell growth	EGFR downregulation	1/37	0.002549	0.049769	0.094675	9/22	0.001732	*SH3GL2*
R-HSA-163359	PKA activation and energy transform	Glucagon signaling in metabolic regulation	1/40	0.002756	0.0537	0.094675	2/6	4.72E-04	*ADCY7*
R-HSA-432720	Cell growth	Lysosome vesicle biogenesis	1/43	0.002963	0.057615	0.094675	2/8	6.30E-04	*SH3GL2*
R-HSA-111933	Calcium and sodium	Calmodulin induced events	1/43	0.002963	0.057615	0.094675	1/23	0.001811	*ADCY7*
R-HSA-111997	Calcium and sodium	CaM pathway	1/43	0.002963	0.057615	0.094675	1/24	0.00189	*ADCY7*
R-HSA-379726	Mitochondria	Mitochondrial tRNA aminoacylation	1/47	0.003238	0.062811	0.094675	1/21	0.001654	*CARS2*
R-HSA-111996	Calcium and sodium	Ca-dependent events	1/48	0.003307	0.064106	0.094675	1/27	0.002126	*ADCY7*

### 3.2 The levels of tryptophan catabolites were associated with DRE and correlated with the treatment outcome in patients receiving add-on multivitamin therapy

We recruited 32 AED-resistant epilepsy patients and 29 control participants. The demographic characteristics are shown in Table 4. Sex was not significantly different between the patients and the controls, but age was older in epilepsy patients than in controls (41.72 ± 10.24 vs. 32.86 ± 12.09, *p* = 0.003).

**Table 4:** The demographic characteristics of DRE patients and controls.

Characteristics	DRE patients	DRE patients after add-on treatment	Control	Comparison							
	Mean ± SD	Mean ± SD	Mean ± SD	*t*/χ2^1^	95% CI^1^	*p* ^1^	*p* ^†1^	*t*/χ^2 2^	95% CI^2^	*p* ^2^	*p* ^†2^
Sex (male, %)	11 (34.4)	–	13 (44.8)	0.67		0.404					
Age, years	41.72 ± 10.24	–	32.86 ± 12.09	3.10	(3.13–14.58)	0.003^*^					
TRP, ppb	11,623.36 ± 5,001.65	11,325.99 ± 2,763.75	19,202.67 ± 9,371.02	−3.88	(−11,518.94 – –3,639.68)	<0.001^*^	<0.001^*^	−4.31	(−11,595.35 – –4,158.01)	<0.001^***^	0.001^***^
Serotonin pathway											
Serotonin, ppb	10.49 ± 7.74	12.44 ± 8.24	46.95 ± 35.06	−3.79	(−56.03– –16.88)	0.001^*^	0.003^*^	−3.65	(−53.80 – –15.21)	0.001^***^	0.018^*^
5-HTP, ppb	3.10 ± 0.21	4.16 ± 0.13	3.59 ± 0.38	−6.15	(−0.6 – –0.33)	<0.001^*^	<0.001^*^	7.57	(0.41–0.72)	<0.001^***^	<0.001^***^
Serotonin/TRP	0.0007 ± 0.0008	0.0011 ± 0.0007	0.0044 ± 0.0082	−2.42	(−0.01 – 0.00)	0.022^*^	0.046*	−2.15	(−0.01 – 0.00)	0.040^*^	0.182
5-HTP/TRP	0.0044 ± 0.0017	0.0004 ± 0.0001	0.0018 ± 0.0016	0.76	(−0.00 – 0.01)	0.450	0.372	−0.83	(0.00–0.00)	0.408	0.691
Serotonin/5-HTP	3.2081 ± 2.0485	2.9768 ± 1.9613	12.5720 ± 9.8052	−3.77	(−14.4 – –4.31)	0.001^*^	0.003^*^	−3.98	(−14.52 – –4.67)	<0.001^***^	0.008^**^
Indole pathway											
IAA, ppb	170.61 ± 114.23	449.42 ± 294.70	437.30 ± 446.96	−3.12	(−440.83 – –92.54)	0.004^*^	0.006^*^	0.11	(−201.61 – 225.86)	0.910	0.747
IPA, ppb	57.58 ± 49.35	102.43 ± 95.19	135.17 ± 111.64	−3.38	(−124.09 – –31.08)	0.002^**^	0.003^**^	−1.11	(−91.87 – 26.41)	0.271	0.304
IAA/TRP	0.0212 ± 0.0279	0.0386 ± 0.0223	0.0254 ± 0.0208	−0.67	(−0.02 – 0.01)	0.503	0.682	2.21	(0.00–0.03)	0.031^*^	0.029^*^
IPA/TRP	0.0237 ± 0.0734	0.0097 ± 0.0105	0.0116 ± 0.0207	0.84	(−0.17 – 0.04)	0.403	0.341	−0.39	(−0.01 – 0.01)	0.695	0.890
Kynurenine pathway											
KYN, ppb	680.67 ± 496.48	662.64 ± 272.83	1,220.18 ± 813.27	−3.09	(−891.22– –187.79)	0.003^*^	0.006^*^	−3.46	(−884.20 – –230.89)	0.001^**^	0.006^**^
KYNA, ppb	11.00 ± 3.59	12.14 ± 3.34	14.85 ± 6.34	−2.95	(−6.46 – –1.24)	0.004^*^	0.007^*^	−1.99	(−5.45 – 0.03)	0.053	0.087
KYN/TRP	0.0634 ± 0.0455	0.0631 ± 0.0426	0.0606 ± 0.0238	0.29	(−0.02 – 0.02)	0.773	0.720	0.27	(−0.02 – 0.02)	0.792	0.808
KYNA/KYN	0.1213 ± 0.2132	0.0283 ± 0.0450	0.0522 ± 0.1383	1.52	(−0.02 – 0.16)	0.135	0.080	−0.81	(−0.08 – 0.04)	0.423	0.784

TRP, tryptophan; 5-HTP, 5-hydroxytryptophan; IAA, 3-indole acetic acid; IPA, 3-indolepropionic acid; KYN, kynurenine; KYNA, kynurenic acid. *p < 0.05; **p < 0.01; ***p < 0.001. p† adjusted by age. ^1^The comparison of TRYCATs between DRE patients and controls. ^2^The comparison of TRYCATs between DRE patients after multivitamin supplementation treatment and controls.

To investigate whether TRYCATs were associated with epilepsy, we compared the level of TRYCATs between groups (Table 4). The results showed that DRE patients had significantly lower levels of TRP than the controls (11,623.36 ± 5,001.65 vs. 19,202.67 ± 9,371.02, *p* < 0.001). Regarding the serotonin pathway, DRE patients had significantly lower levels of serotonin (10.49 ± 7.74 vs. 46.95 ± 35.06, *p* = 0.001) and 5-HTP (3.10 ± 0.21 vs. 3.59 ± 0.38, *p* < 0.001) than the controls. We further calculated the ratio of serotonin/TRP, 5-HTP/TRP, and serotonin/5-HTP because TRP is an essential amino acid obtained from the diet. The results showed that the ratios of serotonin/TRP (*p* = 0.022) and serotonin/5-HTP (*p* = 0.001) were significantly lower in DRE patients than in controls. Regarding the indole pathway, DRE patients had significantly lower levels of IAA (170.61 ± 114.23 vs. 437.30 ± 446.96, *p* = 0.004) and IPA (57.58 ± 49.35 vs. 135.17 ± 111.64, *p* = 0.002). Regarding the KYN pathway, DRE patients also had significantly lower levels of KYN (680.67 ± 496.48 vs. 1,220.18 ± 813.27, *p* = 0.003) and KYNA (11.00 ± 3.59 vs. 14.85 ± 6.34, *p* = 0.004). In addition, these results were still consistent after adjustment for age.

**Figure 4:**
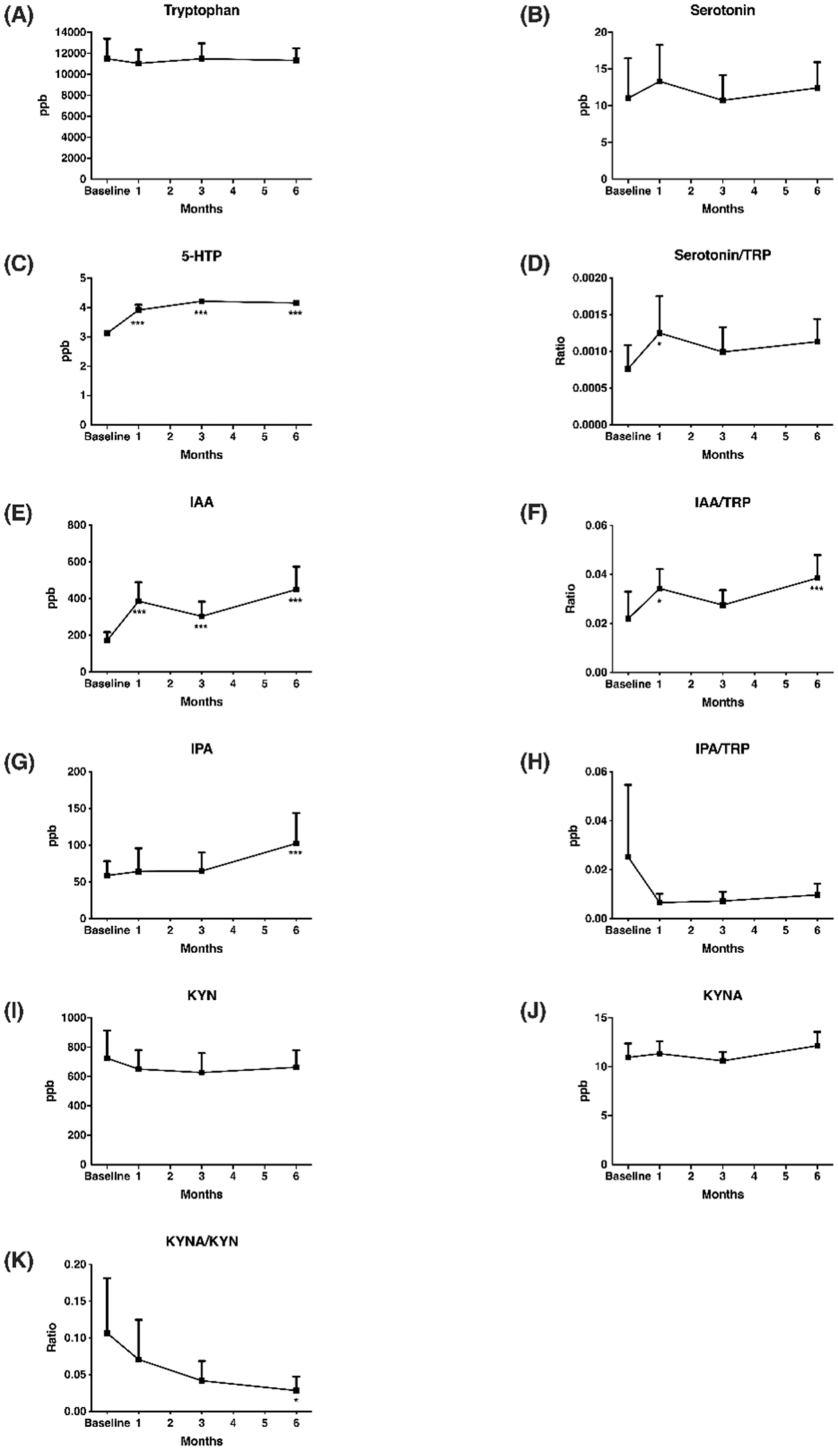
Change of TRYCATs in the 6 months of multivitamin supplementation. **(A)** Tryptophan; **(B)** Serotonin; **(C)** 5-HTP; **(D)** Serotonin/TRP; **(E)** IAA; **(F)** IAA/TRP; **(G)** IPA; **(H)** IPA/TRP; **(I)** KYN; **(J)** KYNA; **(K)** KYNA/KYN. *Y* axis represented TRYCATs levels or ratio, and *x* axis displayed time (by month). Significance were calculated by GEE, comparing baseline and after vitamin supplementations. TRP, tryptophan; 5-HTP, 5-hydroxytryptophan; IAA, 3-indole acetic acid; IPA, 3-indolepropionic acid; KYN, kynurenine; KYNA, kynurenic acid. *p < 0.05, ***p < 0.001.

After multivitamin supplementation, the levels of TRYCATs at baseline and after 1, 3, and 6 months are presented in Figure 4. The levels of 5-HTP, IAA, and IPA during follow-up were significantly higher than at baseline. Compared to the controls, the patients with epilepsy after 6 months of multivitamin supplementation had significantly lower levels of TRP (11,325.99 ± 2,763.75 vs. 19,202.67 ± 9,371.02, *p* < 0.001), serotonin (12.44 ± 8.24 vs. 46.95 ± 35.06, *p* = 0.001), and KYN (662.64 ± 272.83 vs. 1,220.18 ± 813.27, *p* = 0.001) (Table 4). Interestingly, patients after multivitamin supplementation had significantly higher 5-HTP levels (4.16 ± 0.13 vs. 3.59 ± 0.38, *p* < 0.001) than the controls.

To investigate whether the change levels of TRYCATs were correlated with the treatment outcome of add-on multivitamin in patients, correlations between changes in seizure frequency and changes in TRYCAT levels were performed (Table 5). The results showed that the change in seizure frequency was significantly negatively correlated with the change in the levels of TRP, serotonin, serotonin/TRP, serotonin/5-HTP, and KYN, but positively correlated with the change in the levels of KYNA/KYN, IPA, IAA/TRP, and IPA/TRP.

**Table 5:** Correlations between changes in seizure frequency and changes in TRYCATs levels in patients with epilepsy.

Changes in TRYCATs levels	Delta frequency	
	*r*	*p*
Delta TRP	−0.274	0.013*
Serotonin pathway		
Delta serotonin	−0.229	0.040*
Delta 5-HTP	−0.143	0.201
Delta serotonin/TRP	−0.223	0.046*
Delta 5-HTP/TRP	0.179	0.068
Delta Serotonin/5-HTP	−0.252	0.023*
Indole pathway		
Delta IAA	0.112	0.320
Delta IPA	0.242	0.033*
Delta IAA/TRP	0.218	0.048*
Delta IPA/TRP	0.307	0.005*
Kynurenine pathway		
Delta KYN	−0.280	0.011*
Delta KYNA	−0.124	0.271
Delta KYN/TRP	−0.177	0.112
Delta KYNA/KYN	0.294	0.008*

TRYCATs, tryptophan catabolites; TRP, tryptophan; 5-HTP, 5-hydroxytryptophan; IAA, 3-indole acetic acid; IPA, 3-indolepropionic acid; KYN, kynurenine; KYNA, kynurenic acid. *p < 0.05.

## 4 Discussion

To our knowledge, this was the first clinical study to clarify the role of TRYCATs in DRE patients by leveraging the enrichment analysis of genomic analysis from the integration of Taiwan Biobank and clinical outpatients. In the current study, we obtained candidate genes implicated in mechanisms against epilepsy using the GWAS approach and found molecular signature profiles, such as TRYCATs, using enrichment analysis of genomic data. In addition, we validated the results and found a significantly different pattern of TRYCATs between the DRE patients and controls. We also found that changes in the balance of the TRYCATs pathway were noted in DRE patients treated with 6-month multivitamin supplementation. Furthermore, the change levels of TRYCATs were correlated with seizure frequency in the DRE patients receiving multivitamin supplementation. Thus, our study demonstrated the clinical utility of the GWAS approach in epilepsy. Moreover, the results of this study support that TRYCATs play an important role in epilepsy pathophysiology, and the balance of TRYCATs might be a new therapeutic strategy for epilepsy, especially in DRE patients.

In the current study, we found that some candidate genes from the GWAS approach might be involved in the mechanisms of epilepsy, such as *CSMD1, CARS2, ADCY7,* and *GRIK2.* Previous studies revealed that *CSMD1* may play an important role in cognitive functions, schizophrenia, and Parkinson’s disease (39, 40), affect the ratio between dopamine and serotonin metabolites in cerebrospinal fluid (41), and provide a link between the immune system and neuronal processes (42). For *ADCY7*, previous studies mentioned the effect of *ADCY7* suppression on cAMP signaling during hypoxia (43), while it also showed an important role in heart failure with impaired mitochondrial respiration (44), oxidative stress in Parkinson’s disease (45), and the interaction between oxidative stress and TRP metabolism in neuroinflammation (46). In addition, polymorphisms of *GRIK2* were associated with an increased risk of epilepsy in children (47), while polymorphisms of *CARS2* were associated with severe progressive myoclonic epilepsy and mitochondria-related diseases (48, 49).

Furthermore, to leverage these genomic profiles, we performed enrichment analysis from GWAS data and found functional pathways that might be involved in the pathophysiology of epilepsy, such as neurotransmitter signal transmission, PKA activation, and interleukin signaling. Regarding interleukin signaling, the results of the current study suggested that there was an association between interleukin and epilepsy, as we found interleukin signaling pathways (R-HSA-9008059 and R-HSA-446652) in our gene set, which were referred to as the IL-1 family and IL-37 in the pathway analysis. IL-37b, previously called IL1F7, is one of the most recently discovered IL-1 family members and is known to modulate inflammation (50). In the mechanism between cytokines and oxidative stress, evidence has shown that cytokines can activate IDO, catalyzing the degradation of TRP into KYN. Quinolinic acid (QUIN), one of the metabolites of KYN, can stimulate N-methyl-D-aspartate (NMDA) receptors and lead to glutamatergic overproduction. In addition, interleukins can increase ROS production and lead to neuroinflammation (51). A study further showed that patients with epilepsy had higher IL-1 and IL-6 levels than controls and had a decrease in serum IL-1 and IL-6 levels after surgery (52). In addition, we observed the PKA energy conversion-related pathway in the pathway analyses. A study indicated that the expression of glucagon-like peptide 1 (GLP-1) receptors, upstream of PKA signaling, is relatively lower in epilepsy patients than in controls, and the same result was also shown in rat models (53, 54). The lower the GLP-1 level, the worse the diabetic syndrome might be. This phenomenon might be a reasonable explanation for the higher HbA1c values observed in patients with epilepsy from the data of the Taiwan Biobank (5.80 ± 0.87 vs. 5.67 ± 0.76%). Further studies revealed that perampanel, an AED, could affect the phosphorylation of the PKA downstream pathway. A higher phosphorylation ratio of PKA and extracellular signal-regulated kinase 1/2 (ERK1/2) was noted in epilepsy rats using perampanel, which is similar to untreated mice without epilepsy (55). Therefore, the interactions between PKA activation and perampanel may explain why our clinical patients had a lower HbA1c value than the controls, as approximately 60 percent of our DRE patients received long-term (>6 months) perampanel use (7). Regarding the neurotransmitter pathway, our gene set was related to the gamma-aminobutyric acid (GABA) receptor pathway. According to the “neurotransmitter receptors and postsynaptic signal transmission” mechanism in the Reactome database, we found that serotonin modulates the influx of sodium, potassium, and calcium by binding to the 5-hydroxytryptamine receptor 3A (HTR3A) pentamer and further affecting the GABA receptor (56). Our results tried to prove the possible concept of the mechanism by examining the level of serotonin and showed relatively lower serotonin in the DRE patients (10.49 ± 7.74 vs. 46.95 ± 35.06 ppb, *p* = 0.001). In addition, 5-HT3 receptor excitement and antagonism can change the pentylenetetrazol-induced clonic convulsion threshold in a mouse model (57). Although serotonin receptors and postsynaptic signal transmission have been widely discussed in depression, the possible mechanism and clinical application in epilepsy still need to be further confirmed. Taken together, our study revealed potential candidate genes and mechanisms involved in epilepsy and in its related treatment using an integrative genomic approach.

**Figure 5:**
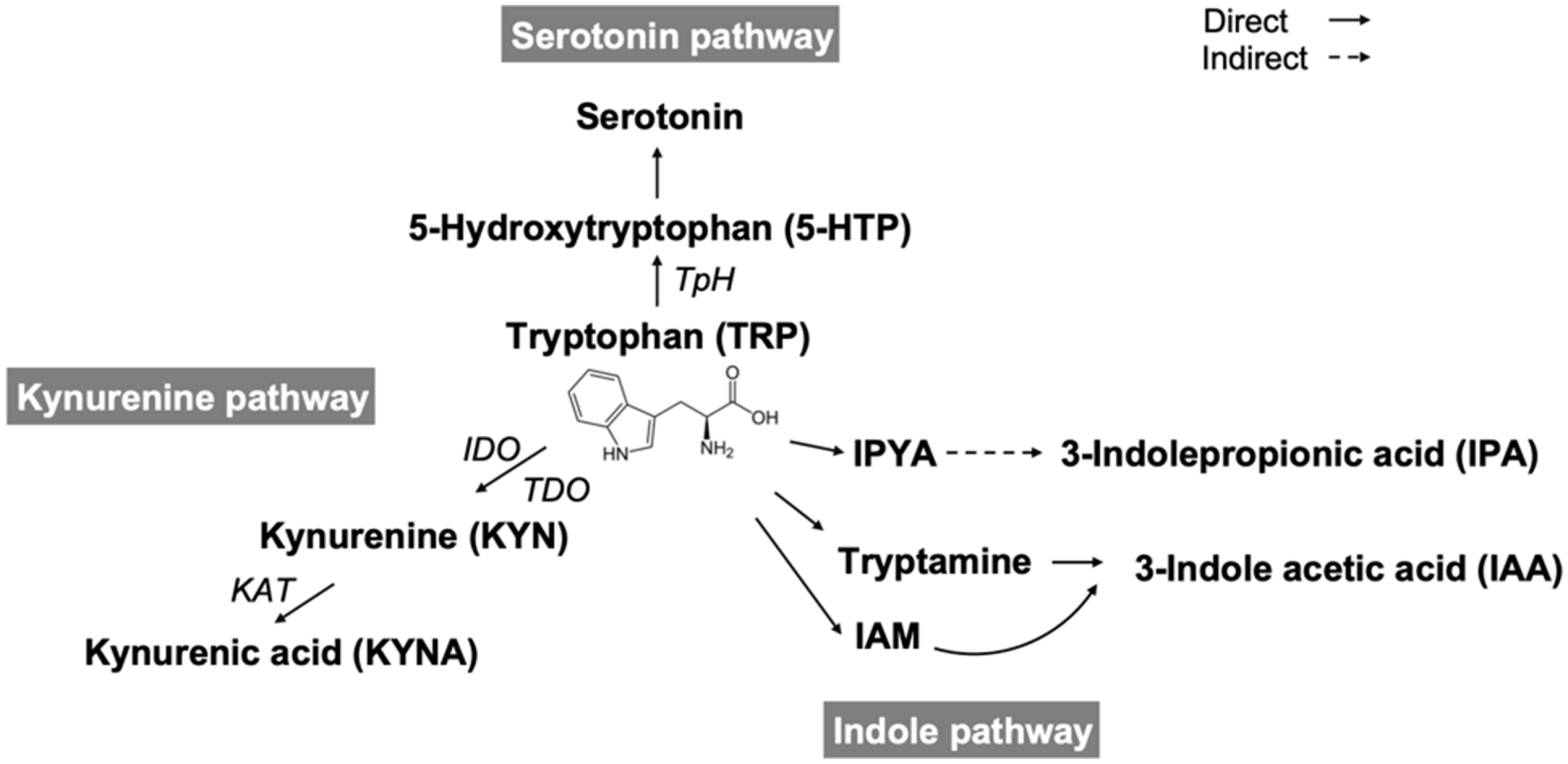
Scheme of tryptophan catabolites. TpH, tryptophan hydroxylase; IDO, indoleamine 2,3-dioxygenase; TDO, tryptophan 2,3- dioxygenase; KAT, kynurenine aminotransferase; IPYA, indole-3-pyruvate; IAM, indole-3-acetamide.

Recent studies have indicated that the balance of the TRP to KYN/serotonin pathway is implicated in inflammation, mood, and cognitive function. Figure 5 shows the scheme of TRYCATs pathway. Furthermore, the TRP-KYN pathway is an inflammatory marker (58–61). This pathway of TRP to KYN/serotonin generates a range of metabolites that are involved in various medical conditions, such as inflammation, the immune response, and several central nervous system (CNS) disorders, including depression and diseases associated with neurodegeneration (58–61). The essential amino acid TRP is obtained mainly from the diet, and several key enzymes are involved in the TRP-KYN metabolic pathway as cofactors, such as pyridoxal phosphate (PLP) (the active form of vitamin B6). TRP and its metabolite KYN cross the blood–brain barrier and modulate CNS diseases and treatment responses. The KYN pathway (or KYN/TRP ratio) even serves as an inflammatory biomarker for treatment-resistant bipolar depression (58). TRP and KYN are degraded to KYNA in astrocytes and to quinolinic acid in microglia to affect NMDA receptors in a pharmacologically opposite fashion; thus, the KYN pathway can be a therapeutic target in cognitive and neurodegenerative disorders (62). Previous studies showed that reductions in serum quinolinic acid and KYN were correlated with AED levels in the periphery and that TRP levels tended to be lower in both the cerebrospinal fluid and serum of seizure patients than in those of nonepileptic subjects (63). Moreover, changes in the TRP and KYN catabolite pathways in the blood were noted in children treated with a ketogenic diet for refractory epilepsy (59). In our current study, we found that DER patients had significantly different levels of TRYCATs from the controls, including the serotonin pathway (TRP, serotonin, 5-HTP, serotonin/TRP, and serotonin/5-HTP), indole pathway (IAA and IPA), and KYN pathway (KYN and KYNA). Taken together, although little is known about whether the balance of the TRP to KYN/serotonin pathway is associated with epilepsy itself and influences the outcomes of vitamin supplementation during AED therapy in patients with epilepsy, further study about TRYCATs focusing on mechanisms and possible effect of therapeutic action is still needed.

TRYCATs play crucial role in the neuroimmune interface of epilepsy. Inflammatory cytokines such as IL-1 and IL-6, often elevated in patients with DRE, activate indoleamine 2,3-dioxygenase (IDO), diverting TRP metabolism from serotonin synthesis toward the KYN pathway (51, 52). This shift results in reduced serotonin levels and increased production of downstream metabolites such as quinolinic acid (a neurotoxic NMDA receptor agonist) and KYNA (a neuroprotective NMDA receptor antagonist) reflecting a dynamic bidirectional regulation within the TRYCATs pathway (59, 62). Our findings of reduced serotonin, KYN, IPA, and IAA levels in DRE patients support this imbalance. Furthermore, the significant correlation between changes in TRYCATs and seizure frequency following multivitamin supplementation suggests that restoration of cofactor availability (e.g., vitamin B6, D, CoQ10) may modulate TRYCAT flux toward neuroprotective outputs (7, 64–66). This mechanistic insight highlights the potential of TRYCAT modulation as a therapeutic strategy.

Accumulating evidence has suggested that vitamin supplementation can influence the balance of the TRP to KYN/serotonin pathway (64, 66, 67), and our results were also consistent with previous reports. For vitamin B6, the change in vitamin level and change in TRP metabolites (TRP, 5-HTP, and KYN) had a significant negative correlation in DRE patients in our data (data not shown). According to previous studies, the active form of PLP could mediate the pathway of 5-HTP to serotonin, TRP to indoles, KYN to KYNA, and other pathways in TRP metabolism (68–70), which was consistent with our findings. For vitamin D, a study revealed that the serum vitamin D level increased significantly and KYN increased at the same time in attention deficit/hyperactivity disorder (ADHD) children receiving 2 months of vitamin D supplementation (66), while we also found a positive correlation with KYNA in DRE patients. Vitamin E has been found to normalize brain serotonin and showed a decrease when serotonin reuptake inhibitors were used (71–73). Vitamin E has also shown the effect of antioxidants on inflammation and on TRP metabolic pathways by mediating the IDO pathway (67, 74, 75). For coenzyme Q10, a recent study showed the effect of coenzyme CoQ10 supplementation on serotonin levels in platelets from fibromyalgia patients and showed the improvement of depressive symptoms (65), while another study revealed the effect of coenzyme Q10 on chronic unpredictable mild stress-induced alterations in hippocampal TRP, serotonin, and KYN concentrations and the KYN/TRP ratio (60). Additionally, a study tried to control seizures by adding TRP to epilepsy children, but it was not effective (76). On the other hand, our previous study demonstrated the effectiveness of vitamin supplementation in DRE patients (24). In our current study, we further demonstrated that DRE patients had significantly increased levels of 5-HTP, IAA, and IPA after receiving multivitamin supplementation. Furthermore, the change in the levels of TRYCATs was correlated with the change in seizure frequency in DRE patients with multivitamin supplementation. Taken together, whether TRYCATs are involved in the pathophysiology of epilepsy and in the therapeutic mechanism of vitamin supplementation in DER patients still needs to be clarified.

While prior research has explored the involvement of TRYCATs in neurological conditions including depression, anxiety, and neuroinflammation, few studies have addressed their role in epilepsy (62, 77–79). Reduced peripheral levels of TRP and KYN, along with altered KYNA/QUIN ratios, have been observed in seizure disorders and in children treated with ketogenic diets for refractory epilepsy (59, 80). These findings suggest the contribution of immune-mediated TRP degradation via the IDO pathway to the pathogenesis of epilepsy. However, earlier studies lacked integration with genetic data or analysis of treatment responsiveness, indicating the need for more comprehensive investigations in further studies. In the current study, we extended our investigation by validating the differential expression of TRYCAT metabolites in clinical samples from patients with DRE. This approach more accurately reflects the study’s focus on using GWAS enrichment results to explore a hypothesized mechanism of TRYCATs metabolic dysregulation in epilepsy, rather than directly establishing one-to-one causal relationships between genetic variants and individual metabolites. This two-stage design represents a widely adopted strategy in translational research (81, 82). This bidirectional framework—from genotype to pathway to metabolite—aligns with emerging paradigms in biomarker discovery and supports the existence of a gene-to-metabolite axis involving inflammation-induced shifts in TRYCAT metabolism, which may contribute to seizure susceptibility in DRE. Taken together, these results suggest the TRYCAT pathway’s potential as both a biomarker and a target for therapeutic intervention in epilepsy management.

There were some limitations of the present study. The first limitation was that the sample size of the DRE patients in this single-site study was relatively small because of the low prevalence of refractory epilepsy. Nevertheless, power analysis using G*Power 3.1 indicated that our sample size (in the TRYCATs analysis, *n* = 32 vs. *n* = 29) has 75–80% power to detect medium effect sizes (*d* = 0.6) at alpha = 0.05. The second limitation was the lack of a placebo group in patients with epilepsy who did not receive add-on multivitamin supplementation due to the high technological and pharmaceutical requirement to develop a placebo capsule. The third limitation was the lack of profiles of correlations of AED concentrations and levels of TRYCATs because we did not obtain the AED concentrations. Moreover, we carefully reviewed seizure frequency and EEG results to evaluate the consistency at each time point evaluation. The fourth limitation was that the levels of TRYCATs were measured in the peripheral nervous system but not in the central nervous system. The fifth limitation was that we focused on DRE patients in the current study, and thus the findings could not be extrapolated to drug-sensitive patients with epilepsy. As a sixth limitation, due to the relatively small sample size and unmeasured factors in the clinical validation cohort, we adjusted only for age rather than incorporating the top PCA-derived covariates when comparing TRYCAT levels between patients with epilepsy and controls. The results remained significant and consistent even after age adjustment. Thus, a longitudinal study with a large sample size is necessary to elucidate the levels of TRYCATs and treatment outcomes in patients with epilepsy.

## 5 Conclusion

Our study demonstrated the clinical utility of the GWAS approach in epilepsy. This was the first clinical study to clarify the role of TRYCATs in DRE patients by leveraging the enrichment analysis of genomic analysis, while a significantly different pattern of TRYCATs existed in DRE patients compared to controls. Our novel results suggest that the TRYCAT pathway plays an important role in the pathophysiology of epilepsy and is involved in multivitamin-mediated physiological alterations in DRE patients. Therefore, balance of the TRYCAT pathway could be a mediator of a shared mechanism of epilepsy and the therapeutic action of medications, such as multivitamin supplementation. Furthermore, the balance of TRYCATs might be a new biomarker and therapeutic strategy for epilepsy, especially in DRE patients.

## Data Availability

The original contributions presented in the study are included in the article/Supplementary material, further inquiries can be directed to the corresponding author/s.

## Glossary

DRE: Drug-resistant epilepsy
GWAS: Genome-wide association study
TRYCATs: Tryptophan catabolites
AEDs: Antiepileptic drugs
HRQoL: Health-related quality of life
SNPs: Single nucleotide polymorphisms
PCA: Principal component analyses
LD: Linkage disequilibrium
IBD: Identity-by-descent
HWE: Hardy–Weinberg equilibrium
MAF: Minor allele frequency
SE: Standard error
EM: Expectation maximization
GATES: Gene-based Association Test using Extended Simes procedure
FDR: False discovery rate
TRP: Tryptophan
5-HTP: 5-hydroxytryptophan
KYN: Kynurenine
KYNA: Kynurenic acid
IPA: 3-indoleperopionic acid
IAA: 3-indole acetic acid
LC–MS/MS: Liquid chromatography–tandem mass spectrometry
ANOVA: One-way analysis of variance
GEE: Generalized estimating equations
BMI: Body mass index
BAI: Body adiposity index
WBC: White blood cell
RBC: Red blood cell
Hb: Hemoglobin
Hct: Hematocrit
PLT: Blood platelet
HbA1c: Glycated hemoglobin A1c
ACglucose: Glucose ante cibum
HDL: High-density lipoprotein cholesterol
LDL: Low-density lipoprotein cholesterol
TG: Triglyceride
AST: Aspartate aminotransferase
ALT: Alanine aminotransferase
AFP: Alpha-fetoprotein
*γ*-GT: Gamma-glutamyl transpeptidase
BUN: Blood urea nitrogen
microALB: Microalbumin
QUIN: Quinolinic acid
NMDA: N-methyl-D-aspartate
GLP-1: Glucagon-like peptide 1
ERK1/2: Extracellular signal-regulated kinase ½
GABA: Gamma-aminobutyric acid
HTR3A: 5-hydroxytryptamine receptor 3A
CNS: Central nervous system
PLP: Pyridoxal phosphate
TpH: Tryptophan hydroxylase
IDO: Indoleamine 2, 3-dioxygenase
TDO: Tryptophan 2, 3- dioxygenase
KAT: Kynurenine aminotransferase
IPYA: Indole-3-pyruvate
IAM: Indole-3-acetamide
ADHD: Attention deficit/hyperactivity disorder
