# Supplementary material for: Leveraging the enrichment analysis from a genome-wide association study against epilepsy—focusing on the role of tryptophan catabolites pathway in patients with drug-resistant epilepsy
Source: Front Nutr. 2025 Aug 6;12:1539145. doi: 10.3389/fnut.2025.1539145 (PMC12364821; doi:10.3389/fnut.2025.1539145)
Supplement: Supplementary file 1 [file Data_Sheet_1.pdf]

Table S1. Principal component analysis of clinical indices.

Variables	Principal components						
	Factor 1 (Bone)	Factor 2 (Obesity)	Factor 3 (Blood cell)	Factor 4 (Blood pressure)	Factor 5	Factor 6	Factor 7
**% of variance explained**	23.205	19.496	10.742	7.439	6.054	5.789	4.976
**Z score**	0.987*	-0.002	0.026	0.021	-0.018	0.054	0.019
**Age-matched**	0.976*	0.000	0.055	0.044	-0.009	0.081	0.023
**Young-adult**	0.975*	0.004	-0.064	-0.112	-0.081	-0.082	-0.022
**T-score**	0.975*	0.006	-0.062	-0.110	-0.081	-0.081	-0.026
**Stiffness index**	0.973*	0.060	0.127	-0.008	0.005	0.021	-0.072
**Hip circumference**	0.027	0.944*	-0.006	0.107	0.041	-0.007	0.034
**Waist circumference**	-0.013	0.871*	0.144	0.209	0.164	0.121	0.106
**Body weight**	0.041	0.851*	0.250	0.245	0.162	0.100	-0.026
**Body fat rate**	0.026	0.621*	-0.354	-0.124	-0.106	-0.241	0.305
**Hematocrit**	0.003	0.138	0.848*	0.133	0.184	0.044	0.064
**Hemoglobin**	0.014	0.171	0.815*	0.245	0.259	0.044	0.075
**Albumin**	0.018	-0.019	0.623*	-0.125	-0.162	-0.018	0.065
**Bilirubin**	0.061	-0.108	0.420*	0.043	-0.082	0.041	-0.389

**Systolic pressure**	-0.077	0.150	0.003	0.904*	0.079	0.112	0.102
**Diastolic pressure**	-0.046	0.223	0.164	0.874*	0.077	0.003	0.061
**Gamma-glutamyl transpeptidase**	-0.075	0.122	0.028	0.007	0.816*	0.049	0.089
**Aspartate aminotransferase**	-0.043	0.060	0.067	0.122	0.798*	0.018	-0.024
**Blood urea nitrogen**	-0.016	-0.033	-0.038	0.051	0.027	0.861*	0.148
**Creatinine**	0.019	0.093	0.101	0.053	0.044	0.847*	-0.116
**Low-density lipoprotein**	-0.010	0.025	0.280	0.025	-0.145	-0.009	0.768*
**Glycated hemoglobin A1c**	-0.015	0.122	-0.063	0.197	0.232	0.074	0.505

*Factor loading > 0.4.

Table S2. Principal component analysis of comorbidities.

	Principal components								
**Variables**	**Factor 1**	**Factor 2 (Three-Hypers)**	**Factor 3**	**Factor 4**	**Factor 5**	**Factor 6**	**Factor 7**	**Factor 8**	**Factor 9**
**% of variance explained**	9.252	7.34	5.923	5.515	5.217	4.887	4.849	4.77	4.626
**Schizophrenia**	0.741*	-0.078	0.075	0.060	-0.008	0.259	0.038	-0.004	-0.020
**Parkinson's disease**	0.721*	0.026	0.061	0.118	-0.065	-0.146	-0.077	-0.013	0.067
**Bipolar disorder**	0.422*	0.042	0.134	-0.208	0.389	0.377	0.160	0.042	-0.013
**Hyperlipidemia**	0.073	0.704*	0.021	0.047	0.090	-0.044	-0.020	0.174	-0.124
**Diabetes**	-0.040	0.664*	0.020	-0.073	-0.051	-0.013	-0.043	-0.189	-0.035
**Hypertension**	-0.058	0.563*	0.000	0.205	0.043	0.064	0.253	0.193	0.200
**Asthma**	-0.110	-0.151	0.589*	0.120	-0.011	-0.107	0.103	0.043	-0.185
**Dementia**	-0.055	0.080	0.586*	-0.208	-0.041	0.086	0.194	-0.099	0.223
**Paroxysmal hemicrania**	0.286	0.081	0.545*	0.073	0.093	-0.021	-0.128	-0.050	0.038
**Depression**	0.233	0.078	0.459*	0.023	0.086	0.052	-0.141	0.125	-0.146
**Gastroesophageal reflux**	0.038	0.000	0.023	0.627*	-0.031	0.188	0.055	0.057	-0.135
**Irritable bowel syndrome**	0.251	0.060	-0.031	0.588*	0.013	-0.233	0.111	-0.052	0.145
**Postpartum depression**	0.002	-0.057	-0.022	-0.114	0.838*	-0.077	0.035	0.017	0.025

**Vertigo**	-0.092	0.182	0.142	0.320	0.517*	0.095	-0.064	-0.067	0.068
**Alcoholism/Drug addiction**	0.069	-0.015	-0.043	0.093	-0.019	0.850*	-0.047	-0.032	-0.006
**Cardiomyopathy**	-0.024	0.040	-0.043	0.244	0.083	-0.026	0.645*	0.057	-0.251
**Apoplexy**	-0.086	0.210	0.247	0.023	-0.096	0.094	0.551*	-0.055	0.317
**Arrhythmia**	-0.097	0.223	0.143	0.324	-0.007	0.158	-0.434*	0.078	0.034
**Blind**	0.030	-0.008	-0.029	-0.174	-0.083	0.002	0.057	0.776*	0.172
**Valve heart disease**	-0.041	0.085	0.042	0.224	0.062	-0.026	-0.074	0.586*	-0.168
**Other eye diseases**	-0.129	-0.002	0.170	0.103	-0.027	0.090	0.026	0.028	-0.657*
**Allergic**	-0.195	-0.163	0.208	0.271	0.128	0.161	-0.107	0.122	0.484*

*Factor loading > 0.4.

Table S3. GWAS analyses after adjusting for different covariates in statistical models.

Outcome: Epilepsy			
	**Model 1**	**Model 2**	**Model 3**
**Covariates**	--	Comorbidity Factor 1 (Schizophrenia + Parkinson's disease + Bipolar disorder)	Comorbidity Factor 2 (Three-Hypers)
	**Model 4**	**Model 5**	**Model 6**
**Covariates**	Comorbidity Factor 3 (Asthma + Dementia + Paroxysmal hemicrania + Depression)	Clinical Index Factor 1 (Bone)	Clinical Index Factor 2 (Obesity)
	**Model 7**	**Model 8**	
**Covariates**	Clinical Index Factor 3 (Blood cell)	Neurological and psychological diseases	
